# Physical Activity, Sleep Quality, Physical Fitness, and Academic Performance as Factors Associated with Health-Related Quality of Life in Primary and Secondary School Students from the Canary Islands

**DOI:** 10.3390/healthcare14152263

**Published:** 2026-07-24

**Authors:** Daniel Arriscado-Alsina, Raúl Jiménez-Boraita

**Affiliations:** 1 Department of Specific Didactics, Area of Physical and Sports Education, Universidad de La Laguna, 38200 San Cristóbal de La Laguna, Santa Cruz de Tenerife, Spain; darrisca@ull.edu.es; 2 Faculty of Education Sciences and Humanities, Universidad Internacional de La Rioja (UNIR), 26006 Logroño, La Rioja, Spain

**Keywords:** health-related quality of life, schoolchildren, physical activity, sleep problems, physical fitness, academic performance

## Abstract

**Background/Objectives**: Health-related quality of life is a key indicator of well-being during childhood and adolescence. This study analysed its association with lifestyle-related factors, body composition, physical fitness, academic performance, and sociodemographic variables. **Methods**: A cross-sectional study was conducted with 949 schoolchildren from the Canary Islands, Spain, with a mean age of 11.8 ± 1.78 years. Health-related quality of life, moderate-to-vigorous physical activity, and sleep problems were assessed using questionnaires, while physical fitness and body composition were measured using field-based tests and anthropometric assessments. **Results**: Schoolchildren in the highest health-related quality of life tertile showed higher moderate-to-vigorous physical activity, fewer sleep problems, lower body mass index and waist circumference, better lower-body explosive strength, higher cardiorespiratory fitness, and better academic indicators. The multivariable model explained 27.7% of the variance in health-related quality of life. Sleep problems showed the strongest inverse association (β = −0.352; *p* < 0.001), whereas Primary Education (β = 0.256; *p* < 0.001), perceived academic performance (β = 0.124; *p* < 0.001), moderate-to-vigorous physical activity (β = 0.104; *p* = 0.001), lower-body explosive strength (β = 0.090; *p* = 0.045), and male gender (β = 0.094; *p* = 0.003) were positively associated with health-related quality of life. **Conclusions**: Schoolchildren’s health-related quality of life is closely related to sleep-related difficulties and, to a lesser extent, to movement behaviour, lower-body fitness, perceived academic competence, and sociodemographic factors. These results may inform integrated school-based strategies that combine sleep education, opportunities for daily movement, fitness development, and support for students’ perceived academic competence.

## 1. Introduction

Health-related quality of life (HRQoL) has become a central indicator of health status, as it integrates physical, psychological, and social dimensions from a subjective perspective that places the lived experience of well-being at the core of the analysis [1]. In children and adolescents, its assessment has gained particular relevance in public health, as it enables a better understanding of how young people perceive their well-being and allows the early identification of subgroups in vulnerable situations. This approach is especially pertinent during adolescence, a developmental stage characterised by profound physical, psychological, and social changes that may influence everyday functioning [2]. Longitudinal evidence indicates that HRQoL does not follow homogeneous trajectories, with distinct developmental patterns and clinically relevant declines being identified, particularly among adolescent girls [3].

From a behavioural perspective, HRQoL is closely associated with daily lifestyle behaviours, particularly physical activity and sleep [4]. Physical activity has shown consistent associations with higher global and domain-specific levels of HRQoL in both observational studies and intervention research [5]. Sedentary behaviour and screen time are also relevant lifestyle dimensions for youth health and well-being [6]; however, they were not included among the main behavioural indicators analysed in the present study, which focused on MVPA and sleep problems. Recent reviews have reported consistent associations between poorer sleep quality and lower HRQoL, as well as benefits associated with higher levels of physical activity and sports participation [7].

Beyond self-reported behaviours, physical fitness is a relevant marker of health status during childhood and adolescence. Cardiorespiratory fitness and muscular strength have been linked to lower total and central adiposity and to a more favourable cardiometabolic profile from early ages [8]. Regarding HRQoL, higher levels of cardiorespiratory fitness and muscular strength have been positively associated with different physical, psychological, and social dimensions in children and adolescents [9]. Moreover, longitudinal studies suggest that physical fitness is not only concurrently related to HRQoL but may also predict its future development. In adolescents, both cardiorespiratory fitness and muscular strength have been prospectively associated with higher HRQoL levels over a 24-month period, with a cumulative effect observed when several fitness components are at optimal levels [10].

Several sociodemographic variables have also been consistently linked to HRQoL during childhood and adolescence. In large European samples assessed using the KIDSCREEN-52, boys and girls have been shown to report similar HRQoL levels at younger ages, whereas girls’ HRQoL tends to decline more markedly with age across most dimensions, with significant variability also observed between countries [11]. Systematic socioeconomic inequalities in HRQoL and mental health have also been documented among children and adolescents aged 8 to 18 years, with a higher risk of poorer perceived well-being in less advantaged contexts [12]. The territorial environment may also shape adolescents’ well-being experiences, with differences identified between young people living in urban and rural areas [13]. Finally, studies focused on subjective well-being indicate that gender differences vary across countries and schools, reinforcing the role of the sociocultural and school context in shaping adolescent well-being [14].

Within the school context, academic performance has been consistently associated with HRQoL in children and adolescents. Cross-sectional studies indicate that better academic performance is related to higher scores in psychosocial dimensions such as the school environment, autonomy, and self-perception [15]. Likewise, self-perceived academic performance has been positively associated with HRQoL even after adjustment for sociodemographic and behavioural variables [16]. Recent longitudinal evidence suggests that academic performance during adolescence may predict subsequent HRQoL levels, whereas the reverse association has not shown consistent results, pointing to a possible directionality from academic achievement to perceived well-being [17]. The proposed associations can be interpreted within a biopsychosocial and ecological framework, in which children’s and adolescents’ HRQoL is understood as the result of interactions between biological, behavioural, psychological, social, and contextual factors [18,19]. From this perspective, physical activity, sleep, physical fitness, academic experiences, and sociodemographic conditions should not be viewed as isolated factors, but rather as interrelated dimensions of daily functioning. Physical activity may be associated with HRQoL through several potential pathways, including cognitive, emotional, behavioural, and social mechanisms [20]. In addition, self-determination theory suggests that perceived competence, autonomy, and relatedness are central psychological needs for motivation and well-being, which may help explain the relevance of perceived academic competence and active behaviours in relation to HRQoL [21]. Physical fitness may also be positioned within a developmental pathway linking motor competence, perceived competence, engagement in physical activity, and health-related outcomes [22]. Finally, sleep may represent a key behavioural mechanism connecting daily lifestyle patterns with HRQoL, given its close relationship with emotional regulation, mood, cognitive functioning, and daytime adaptation [23]. These associations may also vary according to gender, educational stage, socioeconomic context, and family or school environments, which may act as moderating factors. Although the present study did not test mediation or moderation models, this theoretical perspective supports the simultaneous examination of behavioural, physical, academic, and sociodemographic indicators when studying HRQoL in school-aged populations.

The Canary Islands represent a particularly relevant setting for studying HRQoL in school-aged populations. They constitute one of the European Union’s outermost regions, a group of territories characterised by geographical remoteness, insularity, territorial fragmentation, and specific socioeconomic constraints [24]. These structural characteristics may shape access to health-promoting environments, organised sport, active commuting opportunities, educational resources, and health-related services. In addition, the Canary Islands are a demographically dynamic territory. According to the Canary Islands Statistics Institute, the archipelago had 2,258,866 inhabitants on 1 January 2025, and 23.5% of the population had been born outside Spain, compared with 19.3% nationally [25]. The region also experiences substantial tourism pressure, with 17.77 million tourists recorded in 2024 [26]. Together with the coexistence of urban, suburban, and rural areas across different islands, these features create a heterogeneous context in which behavioural, academic, physical, and sociodemographic factors related to HRQoL may operate differently from mainland or more territorially homogeneous settings. Generating evidence from this region is therefore important to inform school-based and public health strategies that are sensitive to the specific characteristics of island territories.

Although interest in HRQoL has grown considerably, much of the literature has addressed its associated factors in a fragmented manner. The scarcity of integrated and theoretically informed models estimating the relative contribution of lifestyle behaviours, physical fitness, academic performance, and contextual variables limits a holistic understanding of perceived well-being during childhood and adolescence. From a multidimensional perspective, there is a need to advance towards comprehensive analyses in large school-based samples that consider behavioural, functional, academic, and sociodemographic indicators simultaneously. Therefore, the aim of the present study was to analyse the association between HRQoL and a set of behavioural, functional, academic, and sociodemographic indicators in primary and secondary school students from the Canary Islands. By applying an integrated approach to a large real-world school-based sample, this study seeks to contribute to a more comprehensive understanding of the multidimensional correlates of perceived well-being during childhood and adolescence. Based on previous evidence, we hypothesised that higher levels of moderate-to-vigorous physical activity, better physical fitness, healthier body composition indicators, and more favourable academic performance indicators would be positively associated with HRQoL. Conversely, greater sleep problems were expected to be inversely associated with HRQoL. We also expected sociodemographic variables, particularly gender and educational stage, to be associated with HRQoL, with lower levels anticipated among girls and students in Secondary Education.

## 2. Materials and Methods

### 2.1. Sample and Procedure

The study was conducted in the Autonomous Community of the Canary Islands (Spain), within the socio-educational context, using a cross-sectional, descriptive design with a quantitative approach. The research protocol was reviewed and approved by the Ethics Committee for Research and Animal Welfare of the University of La Laguna (registration no. CEIBA2025-3545). The study was also conducted in accordance with the ethical principles set out in the Declaration of Helsinki, ensuring the well-being of the participating students and the objective treatment of the results throughout the research process.

The project was implemented in 60 schools in the Autonomous Community of the Canary Islands, after obtaining authorisation from the Regional Deputy Ministry of Education to access the participating schools. The schools were evenly distributed between the islands of Tenerife and Gran Canaria, which together account for approximately 82% of the population of the archipelago. On each island, 15 Primary Education schools and 15 Secondary Education schools were included. To promote a balanced and diverse sample composition, a two-stage sampling procedure was applied on each island. In the first stage, schools were stratified according to their socioeconomic and cultural level (low, medium, and high) and were subsequently selected randomly and proportionally to the size of each stratum using proportional allocation. Replacement schools were also established to cover possible refusals; in ten cases, it was necessary to use these replacement schools, maintaining the same socioeconomic and cultural level as the initially selected schools. In the second stage, cluster sampling was applied, considering classroom groups from the fourth year of Primary Education and the second year of Secondary Education as the sampling units. In each selected school, one classroom group was randomly chosen to participate in the study.

Once the participating classroom groups had been defined, all students in these classes were invited to take part in the research through an information sheet and an informed consent form. This form had to be signed by the student’s father, mother, or legal guardian and included information on the aims of the study, the planned assessments, the instruments and tests to be used, the possibility of withdrawing from the study at any time, and the guarantees regarding data protection and confidentiality. In total, 1333 students from the 60 selected sampling units were invited to participate, with an average of 22 participants per group. Of these, 987 provided the corresponding consent, representing a participation rate of 74%. Subsequently, 38 participants were excluded from the analyses because they were unable to complete the physical tests due to injury or illness, did not complete the questionnaires because of language difficulties, or responded in a random or unreliable manner. The final sample consisted of 949 schoolchildren, with a mean age of 11.8 years (SD = 1.78). Although no formal a priori power-based sample size calculation was conducted specifically for the present secondary analysis, the sampling framework was predefined in the institutional protocol of the broader school-based project. The protocol established the inclusion of 60 schools, evenly distributed between Tenerife and Gran Canaria and between Primary and Secondary Education, with one classroom group selected as the sampling unit in each school. This sampling strategy was designed to ensure geographical, socioeconomic, and sociocultural diversity across the educational system. In addition, a sensitivity power analysis was conducted to estimate the minimum detectable effect size with the final analytical sample. For a multiple linear regression model with 12 predictors, assuming α = 0.05, statistical power = 0.80, and N = 949, the study was able to detect a small effect size, expressed as Cohen’s f^2^, of approximately 0.018. This corresponds to an explained variance of approximately 1.8%, supporting the adequacy of the sample size for detecting small but potentially meaningful associations in the present model [27,28].

Data collection was carried out between May and November 2025 by two assessment teams, one on each island, each composed of three graduates in Physical Activity and Sport Sciences. These professionals were selected through a public call and received specific training to perform the assessment tasks. Previously validated instruments for school-aged populations were used to collect the information. All measurements were adapted to the age and characteristics of the students and were carried out in a safe and familiar environment, namely the students’ own school. The data recording process also followed a standardised and systematic protocol to ensure the validity and reliability of the measurements.

A full description of the methodological design, including the general study protocol, school selection procedure, assessment instruments, fieldwork organisation, and human and material resources, is available in the complementary methodological report of the project [29].

### 2.2. Instruments

#### 2.2.1. Health-Related Quality of Life

Health-related quality of life was assessed using the KIDSCREEN-10 questionnaire, which has been validated in European children and adolescents, including the Spanish population [30]. This instrument provides a global measure of physical, psychological, and social well-being based on ten items. In the present study, the total score was transformed into a 0 to 100 scale to facilitate the interpretation of the results, with higher values indicating better health-related quality of life. This score was subsequently categorised into tertiles of the sample distribution to establish three relative levels of HRQoL: low, medium, and high.

#### 2.2.2. Physical Activity

Habitual physical activity was assessed using the Physical Activity Unit 7-item Screener (PAU-7S), validated in Spanish schoolchildren by Schröder et al. [31]. This instrument estimates moderate-to-vigorous physical activity (MVPA) performed during the previous week through seven items that record the frequency and duration of such activity. Based on the responses, the average daily minutes of self-reported MVPA during the previous week were calculated.

#### 2.2.3. Physical Fitness

Physical fitness was assessed using the protocols of the ALPHA-Fitness battery [32], which was designed to evaluate key components of health-related physical fitness. Following the authors’ recommendations, three tests were administered: handgrip dynamometry (kg) to assess handgrip strength; standing long jump (cm) to assess lower-body explosive strength; and the 20-metre shuttle run test to estimate cardiorespiratory fitness, expressed as VO_2_max (mL/kg/min).

#### 2.2.4. Sleep Habits

Sleep patterns and sleep quality were assessed using the Sleep Self-Report (SSR), a questionnaire designed to detect sleep problems in children and adolescents and validated in the Spanish population by Orgilés et al. [33]. The instrument includes three introductory questions and 16 scored items grouped into four dimensions: sleep quality, sleep-related anxiety, bedtime resistance, and sleep routines. Each item is answered using a three-point frequency scale (0–2), with higher scores reflecting a greater presence of sleep-related difficulties. For the analyses, the total score of the 16 scored items of the Sleep Self-Report was used, with higher values indicating a greater presence of sleep problems.

#### 2.2.5. Body Composition

Waist circumference, body weight, and height were measured following the standardised procedures proposed by the International Society for the Advancement of Kinanthropometry (ISAK) [34]. Body mass index (BMI) was calculated from body weight and height.

#### 2.2.6. Socioeconomic Status

Socioeconomic status was estimated using the most recent version of the Family Affluence Scale III (FAS III) [35]. This questionnaire consists of six items assessing the availability of certain material resources and the family’s economic situation, generating a total score ranging from 0 to 13, with higher values indicating greater family affluence.

#### 2.2.7. Academic Performance

Self-reported academic performance was estimated using two questions from the Health Behaviour in School-aged Children study [36], aimed at assessing grades obtained in Mathematics and Spanish Language and Literature. Students were asked to indicate the grade obtained in the previous term in both subjects, selecting one of five categories: fail, pass, good, very good, or excellent. In addition, to assess perceived academic competence in these subjects, students were also asked: “Compared with your classmates, how good are you at the following subjects?”, with five response options ranging from “much worse than them” to “much better than them”. For the analyses, responses for both subjects were recoded on a 1 to 5 scale and averaged, generating two global indicators: grades obtained and perceived grades. In both cases, higher scores indicated better academic performance or a more favourable perception of academic competence.

#### 2.2.8. Sociodemographic Variables

In addition to the variables described above, information was collected on students’ gender and date of birth in order to determine age. Furthermore, according to the location of the schools and the criteria of the Canary Islands Statistics Institute, students were classified by type of territorial environment into three categories: cities, rural areas, and towns and suburbs. This three-category classification was used for the descriptive analyses. For the multivariable linear regression model, the variable was subsequently recoded into a dichotomous format, distinguishing between urban and non-urban environments.

Finally, with the aim of detecting and excluding questionnaires completed in a random, dishonest, or pseudo-random manner, the Oviedo Infrequency Response Scale (INF-OV) was partially used [37]. Although the original version of the instrument consists of 12 items with a five-point Likert response format, in the present study three items were selected, strategically distributed throughout the questionnaire, and adapted to a dichotomous response format (yes/no) to facilitate interpretation. These items referred to obvious and common-sense situations, such as “Have you ever used a bus?” or “Have you ever seen children playing in a park?”, which made it possible to identify incoherent or implausible responses. According to the established quality control criterion, questionnaires were to be excluded when more than one response inconsistent with the expected logic was detected. However, no cases had to be excluded, as no participant exceeded this threshold.

### 2.3. Statistical Analysis

An initial descriptive analysis was conducted for all variables included in the study. Continuous variables were expressed as mean (M) and standard deviation (SD), whereas categorical variables were presented as frequencies and percentages. The normality of the distributions was assessed using the Kolmogorov–Smirnov test and the visual inspection of histograms and Q-Q plots, while homogeneity of variances was examined using Levene’s test. To analyse differences in lifestyle behaviours, body composition indicators, physical fitness, and sociodemographic characteristics according to HRQoL levels, one-way analysis of variance (ANOVA) was used for continuous variables and the χ^2^ test for categorical variables. HRQoL tertiles were used only for descriptive and comparative purposes in order to characterise behavioural, physical, academic, and sociodemographic profiles across relatively low, medium, and high levels of perceived well-being within the study sample. This approach facilitated the interpretation of group differences without imposing external clinical cut-off points, which are not universally established for the transformed KIDSCREEN-10 score in this specific population. Importantly, HRQoL was retained as a continuous variable in the multivariable linear regression model to avoid loss of information and to estimate the independent associations between the study variables and the full HRQoL score. Effect size was estimated using partial eta squared (η^2^p) for parametric comparisons and Cramer’s V for associations between categorical variables.

Additionally, a multivariable linear regression model was estimated with HRQoL (KIDSCREEN-10, 0–100 scale) as the dependent variable. The model simultaneously included sociodemographic variables (gender, educational stage, socioeconomic status, and place of residence), lifestyle and sleep indicators (MVPA and sleep problems), and body composition and physical fitness variables (BMI, handgrip strength, lower-body explosive strength, and cardiorespiratory fitness), as well as academic performance indicators (grades obtained and perceived grades). Educational stage was included as the main developmental and school-related indicator, given that the sample came from two specific school years: the fourth year of Primary Education and the second year of Secondary Education. For the regression model, place of residence was recoded into a dichotomous format, distinguishing between urban and non-urban environments. All participants included in the final sample had valid data for the variables incorporated into the model; therefore, the analyses were conducted using the full sample of 949 schoolchildren. Results were reported using unstandardised coefficients (B), standardised coefficients (β), 95% confidence intervals (95% CI), *p* values, and adjusted R^2^. The assumptions of linearity, normality, and independence of residuals were verified. Zero-order correlation coefficients were used to describe the bivariate association between each predictor and HRQoL. Multicollinearity was assessed using tolerance and variance inflation factor (VIF) values, which were reported for all predictors included in the multivariable regression model. The level of statistical significance was set at *p* < 0.05. All analyses were performed using IBM SPSS Statistics, version 31.0 (IBM Corp., Armonk, NY, USA).

## 3. Results

A total of 949 schoolchildren participated in the study, including 431 girls (45.4%) and 518 boys (54.6%). The mean age of participants was 11.79 ± 1.78 years. The sample was almost equally distributed between the islands of Tenerife (483, 50.9%) and Gran Canaria (466, 49.1%). Regarding educational stage, 455 students (47.9%) were enrolled in Primary Education and 494 (52.1%) in Secondary Education. Most participants attended schools located in towns and suburbs (501, 52.8%) or cities (381, 40.1%), whereas 67 students (7.1%) came from rural areas. The general characteristics of the total sample and by gender, including health-related quality of life, daily moderate-to-vigorous physical activity, sleep problems, body composition, physical fitness, and academic performance indicators, are presented in Table 1.

Table 2 shows differences in lifestyle behaviours and physical fitness indicators according to HRQoL tertiles. Statistically significant differences were observed in MVPA, with progressively higher values from the lowest to the highest HRQoL tertile (*p* < 0.001). Similarly, sleep problems differed significantly across tertiles, with higher scores in the lowest tertile and lower scores in the highest tertile (*p* < 0.001). Regarding body composition, both body mass index and waist circumference showed significant differences, with progressively lower values as HRQoL tertile increased (*p* < 0.001 in both cases). In terms of physical fitness, significant differences were observed in lower-body explosive strength, assessed through the standing long jump, and in cardiorespiratory fitness (estimated VO_2_max), with more favourable values in the highest tertile than in the lowest tertile (*p* = 0.032 and *p* < 0.001, respectively). By contrast, no statistically significant differences were found across HRQoL tertiles in handgrip strength (*p* = 0.883).

Table 3 presents sociodemographic and academic characteristics according to HRQoL tertiles. Significant differences were observed by gender, with a higher proportion of boys in the highest HRQoL tertile and a higher proportion of girls in the lowest tertile (*p* < 0.001). Significant differences were also found according to educational stage, with Primary Education students being more frequently represented in the highest HRQoL tertile, whereas Secondary Education students were more frequently represented in the lowest tertile (*p* < 0.001). Regarding continuous academic and sociodemographic variables, socioeconomic status was significantly higher among participants with higher HRQoL levels (*p* = 0.004). Similarly, both grades obtained and perceived grades showed a progressively increasing pattern, with higher values in the highest HRQoL tertile (*p* < 0.001 in both cases). By contrast, no statistically significant differences were found according to place of residence (*p* = 0.350).

Table 4 presents the multivariable linear regression model associated with HRQoL in schoolchildren. The overall regression model was statistically significant, F (12, 936) = 31.322, *p* < 0.001, explaining 27.7% of the variance in HRQoL. After simultaneous adjustment for all included variables, being enrolled in Primary Education was associated with higher HRQoL levels compared with Secondary Education (β = 0.256; *p* < 0.001), while male gender was associated with higher HRQoL scores compared with female gender (β = 0.094; *p* = 0.003). Similarly, higher daily moderate-to-vigorous physical activity was positively associated with HRQoL (β = 0.104; *p* = 0.001), whereas sleep problems showed the strongest inverse association in the model (β = −0.352; *p* < 0.001). Lower-body explosive strength and perceived grades were also significantly associated with higher HRQoL levels (β = 0.090; *p* = 0.045 and β = 0.124; *p* < 0.001, respectively). The remaining variables included in the model did not show statistically significant associations after multivariable adjustment. Multicollinearity diagnostics indicated that collinearity was not a concern in the model. Tolerance values ranged from 0.335 to 0.972, and VIF values ranged from 1.029 to 2.988, remaining within acceptable thresholds.

## 4. Discussion

The present study examined the association of lifestyle behaviours, physical fitness, academic performance, and sociodemographic factors with HRQoL in a large sample of schoolchildren from the Canary Islands. The multivariable model explained 27.7% of the variance in HRQoL, highlighting the multifactorial nature of perceived well-being during childhood and adolescence. Among the variables analysed, sleep problems showed the strongest inverse association, whereas physical activity, lower-body explosive strength, perceived academic performance, male gender, and being enrolled in Primary Education were positively associated with HRQoL. Given the cross-sectional design of the study, alternative explanations should also be considered when interpreting these findings. Reverse causality cannot be ruled out; for example, students with higher HRQoL may be more likely to engage in MVPA, perceive their academic competence more favourably, and maintain healthier sleep routines. In addition, some of the observed associations may be bidirectional. Sleep problems and lower HRQoL may reinforce each other through daytime fatigue, emotional distress, reduced school engagement, and lower motivation for physical activity. Similarly, perceived academic competence and HRQoL may be mutually related, as students with better perceived well-being may evaluate their academic performance more positively, whereas perceived academic difficulties may be accompanied by lower well-being. Therefore, the present findings should be interpreted as associations rather than causal pathways. Longitudinal and intervention studies are needed to clarify the temporal ordering and potential reciprocal relationships among sleep, MVPA, perceived academic competence, physical fitness, and HRQoL.

Sleep problems emerged as the factor most closely related to HRQoL in the study population, showing a negative association. Adolescence is a critical period for sleep problems due to a combination of biological, environmental, and psychosocial changes that affect sleep regulation and patterns, including alterations in the circadian system and sleep homeostasis. These changes may lead to delayed sleep timing and shorter sleep duration, which are typical features of this developmental stage [38]. The results of the present study are consistent with a recent review concluding that shorter sleep duration and, particularly, poorer sleep quality are associated with lower HRQoL levels [7]. In the same line, a meta-analysis by Bacaro et al. [39] reported that greater sleep quantity and quality in adolescents are associated with better mental well-being, as well as fewer internalising symptoms, such as anxiety and depression, and externalising symptoms, such as addictive and disruptive behaviours.

Several mechanisms may help explain why sleep problems showed the strongest association with HRQoL in the present study. Sleep is closely involved in emotional regulation, cognitive control, attention, and daytime functioning, all of which are central components of perceived well-being during childhood and adolescence. These associations may be partly explained by the fact that poor sleep quality contributes to daytime dysfunction, social exclusion, and reduced self-control, which in turn may increase difficulties in emotion regulation [40]. In addition, a recent systematic review using objective sleep measures, including polysomnography, actigraphy, and Fitbit data, highlighted that sleep disruption and sleep deprivation are related to emotional experience, emotional reactivity, and emotion-regulation capacities in adolescents [41]. Thus, sleep difficulties may be especially relevant to HRQoL because they are closely connected with daily emotional functioning, social experiences, and perceived psychological well-being.

Furthermore, scientific evidence shows that sleep problems during childhood and adolescence may also have negative consequences for cognitive functioning. A meta-analysis including more than 50 studies found that children with sleep problems show deficits in several cognitive domains, including general intelligence, memory, attention, cognitive flexibility, processing speed, and language [42]. These cognitive difficulties may affect school experiences, perceived academic competence, peer relationships, and self-perceived health, all of which are reflected in global HRQoL. Sleep may also interact with other lifestyle behaviours. Insufficient or poor-quality sleep may be associated with lower energy and motivation for physical activity, while a recent systematic review and meta-analysis reported that exercise interventions can improve sleep quality in adolescents [43]. This interaction may partly explain why sleep problems showed a stronger association with HRQoL than more distal indicators such as body composition or some physical fitness components. These findings reinforce the relevance of sleep habits as a key area for school-based health promotion and future intervention research. In this regard, interventions combining educational components with active parental involvement and peer support appear to be more effective in maintaining healthy sleep habits [44].

Physical activity was also positively associated with HRQoL in children and adolescents, supporting evidence of a bidirectional relationship in which higher physical activity may be associated with better HRQoL, while better HRQoL may promote higher levels of physical activity [45]. Reinforcing this idea, intervention studies have reported that physical activity programmes improve multiple HRQoL domains, including physical, psychological, social, and school-related well-being, although the effects tend to be small to moderate [5]. Physical activity may be linked to adolescents’ psychological well-being, self-esteem, and social relationships through multiple interconnected pathways. It may also be associated with lower levels of negative emotions, such as depression and anxiety, both directly through neurobiological effects and indirectly by improving psychological resources such as self-efficacy and social support [46,47]. In addition, social support and self-esteem may act as key mediators in this relationship, as physical activity has been associated with greater life satisfaction and better mental health by strengthening these psychological resources [48]. However, because MVPA was assessed using a self-reported questionnaire, these findings should be interpreted cautiously and should not be considered equivalent to associations based on device-measured physical activity.

The present results also showed a positive association between specific components of physical fitness and HRQoL. Existing evidence indicates that cardiorespiratory fitness and muscular strength are positively associated with several HRQoL domains, including physical well-being, psychological well-being, peer relationships, school performance, and overall HRQoL [9]. In addition, higher baseline levels of cardiorespiratory fitness, muscular strength, and motor competence have been prospectively associated with better HRQoL over time, with cumulative benefits when multiple fitness components are at favourable levels [10]. In the present sample, the descriptive analysis showed significant differences in cardiorespiratory fitness and lower-body explosive strength, with more favourable values among schoolchildren in the higher HRQoL tertiles. However, no significant differences were observed in handgrip strength. In the multivariable regression model, only lower-body explosive strength remained significantly associated with HRQoL. This finding may be explained by shared variance with other factors included in the model, such as MVPA, or because lower-body explosive strength may better reflect motor competence, a factor related to participation in physical and sports activities, as well as to a more favourable perception of physical and psychosocial health during childhood and adolescence [49]. These findings should also be interpreted considering the developmental characteristics of the sample. Participants were between late childhood and adolescence, a period in which biological maturation may influence body size, fat distribution, muscle mass, muscular strength, and cardiorespiratory fitness [50,51]. Therefore, differences in physical fitness and body composition may partly reflect maturational variability rather than behavioural or health-related factors alone. In the present study, body composition was assessed using BMI and waist circumference, and no information on body fat percentage or pubertal status was available. Consequently, the lack of independent associations for BMI and waist circumference, as well as the associations observed for some physical fitness components, should be interpreted with caution.

Although several sociodemographic variables showed associations with HRQoL in the bivariate analyses, only gender, educational stage, and perceived academic performance remained significantly associated in the multivariable model, suggesting that these factors have an independent relationship with HRQoL. Regarding gender, boys reported higher HRQoL levels, in line with the previous literature. Different longitudinal and cross-sectional studies indicate that adolescent boys tend to report better HRQoL than girls, who often experience a decline in HRQoL from childhood to adolescence. This gender gap has been observed across several domains, including physical and psychological well-being, autonomy, and social support, and may reflect a complex interaction of biological, psychological, social, and behavioural factors [52,53]. Although the present study did not assess pubertal variables, menstrual health, or specific mental health indicators, previous research suggests that these factors may partly contribute to gender differences in adolescent HRQoL [54,55]. Mental well-being disparities may also play a relevant role, particularly during the transition to Secondary Education. The physical, emotional, and social changes that occur during this stage may be accompanied by stress, anxiety, distress, and other mental health-related problems, which may especially affect girls and, consequently, their HRQoL [55].

Regarding educational stage, Primary Education students showed significantly higher HRQoL scores than Secondary Education students. Previous studies have shown that HRQoL tends to decline during the transition from childhood to adolescence, with deterioration observed in multiple domains, such as physical well-being, psychological well-being, autonomy, and social support [3,52]. This pattern may be explained by developmental changes characteristic of the transition to adolescence, as well as by the increase in academic, social, and emotional demands during this stage. Developmental changes during this transition include hormonal, metabolic, and neural alterations that influence emotional and cognitive well-being, while behavioural patterns, such as diet, sleep, and physical activity, also play an important role [56,57]. Moreover, as young people grow older, they face new challenges, greater academic demands, and sometimes adverse experiences that may lead to mental health problems such as stress, depression, and anxiety, which are consistently associated with lower HRQoL [58,59].

With regard to academic performance, participants were asked about both the grades obtained in Spanish Language and Mathematics and their perceived competence in these subjects compared with their classmates. In the descriptive analyses, both grades obtained and perceived grades showed more favourable values among schoolchildren in the higher HRQoL tertiles. However, only perceived grades remained significantly associated with HRQoL in the multivariable regression analysis. In this regard, the literature seems to support the idea that adolescents’ perceived academic competence is more strongly associated with their well-being than academic achievement itself. A large longitudinal study showed that although actual competence was a stronger indicator of educational achievement, self-concept was a stronger indicator of well-being over time [60]. Similarly, adolescents who perceive themselves as highly competent show greater intrinsic motivation, better self-regulated learning, and less procrastination, all of which are related to greater well-being [61]. This form of well-being, particularly that linked to personal development and fulfilment, which involves feeling competent and successful, has been positively associated with academic performance, suggesting that the way adolescents perceive their abilities is important for both well-being and achievement [62]. This idea is also consistent with self-determination theory, in which perceived competence, together with autonomy and relatedness, constitutes a basic psychological need for well-being and optimal functioning [21]. In this regard, perceived academic and socioemotional competence have been identified as relevant factors associated with HRQoL, with the former being related to greater school satisfaction and cognitive engagement and the latter predicting mental well-being [63]. These findings suggest that adolescents’ subjective academic competence may play a more relevant role in perceived well-being than objective academic achievement.

Finally, several variables included in the model did not show an independent association with HRQoL after multivariable adjustment, including body mass index, cardiorespiratory fitness, handgrip strength, grades obtained, socioeconomic status, and place of residence. This result does not necessarily imply the absence of a relationship with HRQoL, but rather that their associations may overlap with those of other factors included in the model or may operate indirectly through variables more proximal to perceived well-being. From an applied perspective, these findings suggest that sleep problems, daily MVPA, and students’ perceived academic competence constitute relevant areas to consider when informing school-based strategies aimed at supporting well-being. However, these implications should be interpreted with caution, as the cross-sectional design of the study does not allow determination of whether changes in these factors are followed by subsequent changes in HRQoL. Overall, the results reinforce the role of the school environment as a key context for the development of comprehensive strategies aimed at supporting health-related quality of life in children and adolescents.

More specifically, the present findings may help inform integrated school-based health promotion strategies involving schools, families, and policymakers. At the school level, sleep health could be incorporated into health education, tutoring programmes, and family–school communication, with content focused on regular sleep schedules, sleep hygiene, bedtime routines, and responsible evening screen use. Schools may also promote daily opportunities for MVPA through active breaks, physically active lessons, active recess, extracurricular sport, and active commuting initiatives, while ensuring that these opportunities are inclusive and adapted to different fitness levels. In addition, teachers may support students’ perceived academic competence through autonomy-supportive teaching, constructive feedback, achievable learning goals, and classroom climates that reduce excessive social comparison and promote self-improvement. At the family level, consistent sleep routines, limits on stimulating screen-based activities before bedtime, and encouragement of active leisure may contribute to healthier daily habits. At the policy level, the results support the relevance of coordinated actions between education, health, and sport sectors, particularly during the transition from Primary to Secondary Education, when HRQoL appears to be lower and students may face greater academic, social, and emotional demands.

One of the main strengths of this study lies in its sample size, as it included nearly one thousand schoolchildren from 60 Primary and Secondary Education schools in the Canary Islands. Second, the study adopts a multidimensional approach, integrating lifestyle behaviours, namely physical activity and sleep, field-based physical fitness tests, anthropometric measures, academic variables, and sociodemographic factors within the same model. This approach makes it possible to estimate the relative contribution of different factors associated with adolescent well-being, going beyond previous approaches that have examined these factors in isolation. Finally, data were collected using internationally validated instruments in reference populations, which strengthens the validity of the measurements and facilitates comparison with previous research. In addition, the inclusion of field-based physical fitness tests and anthropometric indicators reduces potential biases associated with the exclusive use of self-reported questionnaires.

Nevertheless, several limitations should be considered when interpreting the results. First, the cross-sectional design prevents the establishment of causal relationships. Therefore, the directionality of the observed associations cannot be determined, and reverse or bidirectional relationships cannot be ruled out. Second, some variables, including physical activity, sleep problems, and perceived academic performance, were assessed using self-report instruments, which may introduce recall or social desirability bias. In particular, MVPA estimates should be interpreted as self-reported physical activity rather than objectively measured activity, as no device-based measures such as accelerometry or pedometers were available. In addition, sedentary behaviour and screen time were not included, despite being relevant components of young people’s movement behaviours and well-being. This limits the ability to determine whether HRQoL is associated specifically with MVPA, with lower sedentary or screen-based behaviours, or with the overall balance between movement behaviours. Future studies should incorporate device-based measures of physical activity and sedentary time, together with specific indicators of recreational and academic screen use.

Third, although the model explained a relevant proportion of the variance in HRQoL, other factors not considered in the present study may also contribute to perceived well-being. Some family characteristics, such as parental education, family structure, and parental occupation, were not assessed. Although socioeconomic status was included through the Family Affluence Scale III, this measure does not fully capture the broader family context in which children’s and adolescents’ well-being develops. Emotional variables were also not included. In addition, the clustered structure of the data was not modelled using multilevel analyses. Although the study included one classroom group per school and focused on individual-level associations, students were nested within schools, and school-level or classroom-level factors may have influenced some of the observed associations. Future studies using similar school-based designs should consider multilevel models to account for clustering and to examine the contribution of school-level contextual factors to HRQoL.

Finally, limitations related to body composition, maturation, timing of assessment, and generalisability should also be acknowledged. Body composition was assessed using BMI and waist circumference, which are useful anthropometric indicators in school-based studies but do not provide direct estimates of body fat percentage, fat-free mass, or body fat distribution. Pubertal status or biological maturation was not assessed, despite its potential influence on body composition, muscular strength, cardiorespiratory fitness, and self-perceived health during late childhood and adolescence. Therefore, associations involving body composition and physical fitness indicators should be interpreted with caution. Data collection also lasted approximately six months, and the analyses did not control for the specific month or season of assessment; consequently, seasonal influences on MVPA, sleep routines, school demands, or perceived well-being cannot be ruled out. Future studies should consider shorter and more homogeneous data collection windows or include timing of assessment as a covariate. Lastly, as the sample was drawn exclusively from the Autonomous Community of the Canary Islands, similar studies should be conducted in other geographical contexts.

## 5. Conclusions

HRQoL in schoolchildren was associated with multiple dimensions of lifestyle behaviours, body composition, physical fitness, the academic context, and sociodemographic characteristics. Descriptive analyses showed a more favourable profile among participants in the highest HRQoL tertile, characterised by higher levels of MVPA, fewer sleep problems, lower body mass index and waist circumference, and better lower-body explosive strength, cardiorespiratory fitness, and academic performance. However, after multivariable adjustment, sleep problems showed the strongest inverse association with HRQoL, whereas MVPA, lower-body explosive strength, perceived grades, male gender, and being enrolled in Primary Education were positively associated with HRQoL.

From an applied perspective, these findings may help inform, but not determine, integrated school-based approaches to HRQoL, with particular attention to factors closely associated with students’ everyday well-being. In this regard, sleep health, daily MVPA, and students’ perceived academic competence may represent relevant areas to consider in future school-based health promotion strategies. However, these implications should be interpreted with caution due to the observational and cross-sectional design of the study, the use of self-reported measures for some variables, the absence of device-based physical activity assessment, the absence of multilevel analyses accounting for the clustered school-based design, and the lack of information on sedentary behaviour, screen time, pubertal status, body fat percentage, and some family-related characteristics. Therefore, the findings should be interpreted as associations and not as evidence of causal relationships. Future longitudinal studies are needed to clarify the directionality of the observed associations, particularly those involving sleep problems, MVPA, perceived academic competence, physical fitness, and HRQoL. In addition, school-based intervention studies should examine whether strategies aimed at improving sleep habits, increasing daily physical activity, and strengthening students’ perceived academic competence are followed by meaningful improvements in HRQoL among children and adolescents.

## Figures and Tables

**Table 1:** General characteristics of the study sample, overall and by gender.

Variable	Total Sample (*n* = 949)	Female (*n* = 431)	Male (*n* = 518)
Age, years	11.79 ± 1.78	11.62 ± 1.78	11.93 ± 1.77
Island, *n* (%)			
Tenerife	483 (50.9)	219 (50.8)	264 (51.0)
Gran Canaria	466 (49.1)	212 (49.2)	254 (49.0)
Place of residence, *n* (%)			
Rural areas	67 (7.1)	28 (6.5)	39 (7.5)
Cities	381 (40.1)	178 (41.3)	203 (39.2)
Towns and suburbs	501 (52.8)	225 (52.2)	276 (53.3)
Educational stage, *n* (%)			
Primary Education	455 (47.9)	227 (52.7)	228 (44.0)
Secondary Education	494 (52.1)	204 (47.3)	290 (56.0)
Health-related quality of life, KIDSCREEN-10	72.05 ± 16.33	68.83 ± 18.04	74.72 ± 14.23
Daily MVPA, min/day	135.51 ± 76.99	120.68 ± 71.61	147.85 ± 79.18
Sleep problems, SSR total	8.20 ± 5.89	9.30 ± 6.10	7.28 ± 5.56
Body mass index, kg/m^2^	20.08 ± 4.07	20.17 ± 3.98	20.00 ± 4.15
Waist circumference, cm	65.35 ± 10.09	64.18 ± 9.59	66.33 ± 10.40
Handgrip strength, kg	19.87 ± 7.83	18.20 ± 5.87	21.26 ± 8.92
Lower-body explosive strength, cm	146.59 ± 28.83	136.70 ± 24.30	154.83 ± 29.73
Cardiorespiratory fitness, mL/kg/min	42.90 ± 5.70	40.86 ± 4.48	44.60 ± 6.04
Grades obtained	3.52 ± 1.16	3.61 ± 1.08	3.45 ± 1.22
Perceived grades	3.36 ± 0.77	3.33 ± 0.72	3.39 ± 0.82

Note. Data are presented as mean ± standard deviation for continuous variables and as *n* (%) for categorical variables. Percentages in the gender-stratified columns are calculated within each gender group.

**Table 2:** Lifestyle behaviours and physical fitness according to tertiles of health-related quality of life.

	HRQoL						*p* Value	Effect Size
	Low (*n* = 338)		Medium (*n* = 318)		High (*n* = 293)			
	M	SD	M	SD	M	SD		
MVPA	113.16	74.49	141.42	75.50	154.88	75.23	<0.001	0.052
Sleep problems	10.30	6.23	7.43	5.47	6.62	5.23	<0.001	0.073
Body mass index (BMI)	20.98	4.30	19.98	3.81	19.15	3.87	<0.001	0.034
Waist circumference	67.19	10.06	65.38	10.09	63.20	9.71	<0.001	0.026
Handgrip strength (kg)	20.21	8.28	19.89	8.50	20.01	8.20	0.883	0.000
Lower-body explosive strength (cm)	143.30	27.26	148.59	29.05	148.23	30.09	0.032	0.007
Cardiorespiratory fitness (mL/kg/min)	40.96	5.38	43.52	5.32	44.47	5.84	<0.001	0.069

Note. Data are presented as mean (M) and standard deviation (SD). Participants were grouped into tertiles of the total KIDSCREEN-10 score. Between-group differences were assessed using one-way analysis of variance (ANOVA). Effect size was estimated using partial eta squared (η^2^p).

**Table 3:** Sociodemographic and academic characteristics according to tertiles of health-related quality of life.

		HRQoL			*p* Value	Effect Size
		Low (*n* = 338)	Medium (*n* = 318)	High (*n* = 293)		
		%	%	%		
Gender	Female	42.50%	30.90%	26.60%	<0.001	0.132
	Male	29.90%	35.70%	34.40%		
Place of residence	Cities	36.00%	31.20%	32.80%	0.350	0.046
	Towns and suburbs	36.50%	34.10%	29.40%		
	Rural areas	26.90%	41.80%	31.30%		
Educational stage	Primary	26.20%	35.80%	38.00%	<0.001	0.200
	Secondary	44.30%	31.40%	24.30%		
		M ± SD	M ± SD	M ± SD		
Socioeconomic status		5.33 ± 2.10	5.54 ± 2.14	5.89 ± 2.19	0.004	0.011
Grades obtained		3.23 ± 1.15	3.56 ± 1.13	3.82 ± 1.11	<0.001	0.043
Perceived grades		3.12 ± 0.77	3.41 ± 0.73	3.59 ± 0.75	<0.001	0.061

Note. Data are presented as percentages for categorical variables and as mean (M) and standard deviation (SD) for continuous variables. Participants were grouped into tertiles of the total KIDSCREEN-10 score. Between-group differences were assessed using the χ^2^ test for categorical variables and one-way analysis of variance (ANOVA) for continuous variables. Effect size was estimated using Cramer’s V for categorical associations and partial eta squared (η^2^p) for parametric comparisons.

**Table 4:** Multivariable linear regression model associated with health-related quality of life.

Variable	B	β	95% CI	*p* Value	Zero-Order r	VIF	Tolerance	Adjusted R^2^
Constant	56.266	—	42.908, 69.623	<0.001	—	—	—	-
Gender (male)	3.065	0.094	1.079, 5.052	0.003	0.180	1.251	0.800	0.277
Educational stage (primary school)	8.355	0.256	5.668, 11.041	<0.001	0.190	2.302	0.434	
Socioeconomic status (FAS III)	0.236	0.031	−0.198, 0.670	0.286	0.128	1.115	0.897	
Place of residence (urban)	−0.007	0.000	−3.508, 3.495	0.997	−0.025	1.029	0.972	
Daily MVPA (min/day)	0.022	0.104	0.009, 0.035	0.001	0.233	1.247	0.802	
Sleep problems (SSR total)	−0.976	−0.352	−1.138, −0.813	<0.001	−0.357	1.174	0.852	
BMI	−0.219	−0.055	−0.506, 0.068	0.134	−0.196	1.744	0.573	
Handgrip strength (kg)	−0.072	−0.035	−0.267, 0.123	0.469	−0.110	2.988	0.335	
Lower-body explosive strength (cm)	0.051	0.090	0.001, 0.101	0.045	0.094	2.632	0.380	
Cardiorespiratory fitness (mL/kg/min)	0.050	0.017	−0.171, 0.271	0.657	0.289	2.017	0.496	
Grades obtained	0.416	0.029	−0.553, 1.385	0.400	0.229	1.606	0.623	
Perceived grades	2.613	0.124	1.211, 4.014	<0.001	0.262	1.506	0.664	

Note. A multivariable linear regression model was estimated to predict health-related quality of life (KIDSCREEN-10, 0–100 scale) in schoolchildren. Results are presented as unstandardised coefficients (B), standardised coefficients (β), 95% confidence intervals (95% CI), *p* values, zero-order correlation coefficients, variance inflation factor (VIF), and tolerance values. The model simultaneously included sociodemographic variables, lifestyle and sleep indicators, physical fitness, adiposity, and academic performance. The overall model was statistically significant, F (12, 936) = 31.322, *p* < 0.001, explaining 27.7% of the variance in HRQoL.

## Data Availability

The data supporting the findings of this study are not publicly available due to ethical and privacy restrictions related to the participation of minors. Anonymised data may be made available from the corresponding author upon reasonable request and subject to approval by the relevant ethics and institutional bodies.

## Abbreviations

ANOVA	Analysis of variance
BMI	Body mass index
CI	Confidence interval
FAS III	Family Affluence Scale III
HRQoL	Health-related quality of life
INF-OV	Oviedo Infrequency Response Scale
ISAK	International Society for the Advancement of Kinanthropometry
MVPA	Moderate-to-vigorous physical activity
PAU-7S	Physical Activity Unit 7-item Screener
SD	Standard deviation
SSR	Sleep Self-Report
VIF	Variance inflation factor
VO_2_max	Maximal oxygen uptake
